# A Phase I/II Clinical Trial of Belinostat (PXD101) in Combination with Doxorubicin in Patients with Soft Tissue Sarcomas

**DOI:** 10.1155/2016/2090271

**Published:** 2016-06-14

**Authors:** Joanna Vitfell-Rasmussen, Ian Judson, Akmal Safwat, Robin L. Jones, Philip Blach Rossen, Maja Lind-Hansen, Poul Knoblauch, Anders Krarup-Hansen

**Affiliations:** ^1^Herlev University Hospital, Herlev Ringvej 75, 2730 Herlev, Denmark; ^2^The Institute of Cancer Research: Royal Cancer Hospital, 123 Old Brompton Road, London SW7 3RP, UK; ^3^Aarhus University Hospital, Nørrebrogade 44, 8000 Aarhus, Denmark; ^4^Onxeo, c/o Symbion, Fruebjergvej 3, 2100 København, Denmark

## Abstract

*Background*. Belinostat is a novel histone deacetylase inhibitor.* Primary Objectives*. Maximum tolerated dose (MTD) and dose limiting toxicities (DLTs) of belinostat (Bel) in combination with doxorubicin (Dox) in solid tumours (phase I) and response rate (RR) in soft tissue sarcomas (phase II).* Methods*. Bel was administered as a 30-minute IV infusion on days 1–5 and on day 5 with Dox. The dose escalation schedule was as follows: cohort 1: Bel 600 mg/m^2^ and 50 mg/m^2^ Dox, cohort 2: Bel 600 mg/m^2^ and 75 mg/m^2^ Dox, cohort 3: Bel 800 mg/m^2^ and 75 mg/m^2^ Dox, and cohort 4: Bel 1000 mg/m^2^ and 75 mg/m^2^ Dox.* Results*. 41 patients were included (25 in phase I, 16 in phase II). Adverse events were fatigue (95%), nausea (76%), and alopecia (63%). There was one DLT, grade 3 rash/hand and foot syndrome. MTD was Bel 1000 mg/m^2^/d and Dox 75 mg/m^2^. Four responses were seen: 2 PR in phase I, RR of 8%; in phase II, 1 PR/1 CR, RR of 13%, and 9 patients (56%) with SD.* Conclusion*. The combination was well tolerated. Response rate was moderate but median time to progression was 6.0 months (95% CI, 1.6–9.7 months) which is superior to some reports of single-agent Dox.

## 1. Introduction

Soft tissue sarcomas (STS) are rare tumours with an annual incidence of 2-3/100,000. Overall STS account for approximately 1% of all malignancies. The median overall survival for patients with metastatic disease is approximately 12 months [1]. There are more than 100 different histological subtypes of STS according to the WHO 2013 classification [2].

Standard curative treatment for high grade STS is wide surgical excision and adjuvant radiotherapy. Neoadjuvant and/or adjuvant chemotherapy can also be considered in selected cases [3]. In rare cases, surgery including amputation is necessary. The primary metastatic site is the lung; however metastases to the bone, liver, and other organs, depending on the site of origin and subtype, are also seen. Chemotherapy is widely used in the treatment of metastatic disease but is mainly palliative [4]. The majority of initial chemosensitive patients develop resistance. Doxorubicin appears to be the most active drug in the treatment of STS with a cumulative response rate in treatment naive patients of approximately 20–30% [5]. The reported response rate for doxorubicin in combination with ifosfamide ranges between 50 and 60% [6], though this combination has not demonstrated improved survival. Other possible treatment options are trabectedin, pazopanib, and paclitaxel in angiosarcoma [7] and a combination of gemcitabine and docetaxel in leiomyosarcoma and undifferentiated pleomorphic sarcoma.

PXD101, currently known as belinostat, is developed by Spectrum Pharmaceuticals and Onxeo (former Topotarget) and belongs to a new class of hydroxamate-type histone deacetylase (HDAC) inhibitors. HDAC inhibitors are inducers of transformed cell differentiation, cell cycle arrest, and apoptosis. They exert their targeted action during posttranslational acetylation of core nucleosomal histones, which affects chromatin structure and leads to the expression of genes associated with cell cycle arrest and tumour suppression. The effects of HDACs are not limited to histone deacetylation. HDACs also act as members of a protein complex to recruit transcription factors to promotor region of genes, including those of tumour suppressors, and they affect the acetylation status of specific cell cycle regulatory proteins [8].

Previous published data with belinostat* in vitro* cancer models have demonstrated a potent antiproliferative and HDAC inhibitory activity and inhibition proliferation of a large variety of human cancer cell lines.* In vivo*, belinostat inhibits growth in human tumour xenografts without apparent toxicity to the host mice [9].

Several other preclinical efficacy models using belinostat alone or in combination with well-established cancer therapeutics have demonstrated tumour growth inhibition and dose dependent increase in animal survival. A strong synergistic effect was obtained in the combination of belinostat and doxorubicin in the sarcoma cell lines SaOS2 and U2OS (*Synergistic In Vitro Growth-Inhibitory Activity of Belinostat/Doxorubicin on Human Sarcoma Cell Lines, nonpublished data*).

Doxorubicin is a widely used anthracycline which acts as a topoisomerase II inhibitor and intercalates into double-stranded DNA and produces structural changes that interfere with DNA and RNA synthesis. DNA-damaging free radical formation may also be important. In soft tissue sarcoma, single-agent doxorubicin 60 to 75 mg/m^2^ is recommended.

Data from previous phase I trials, using belinostat as monotherapy, has established a maximum tolerated dose (MTD) of 1000 mg/m^2^/d for 5 consecutive days repeated in a 3-week schedule for patients with solid tumours and haematological neoplasms. Antitumour effects were observed in a number of different neoplasms, among them 2 STS patients who had prolonged stable disease (SD) for 7 and 14 months, respectively [10, 11].

These data led to the initiation of this trial: a dose escalation phase I/II study of belinostat combined with doxorubicin in patients with solid tumours including an MTD expansion phase II part of belinostat combined with doxorubicin in patients with advanced STS. This study was based on the hypothesis that the addition of belinostat to doxorubicin would improve the antitumour effect, without significantly increasing toxicity.

The primary objectives of this trial were to determine the MTD, DLTs, and the efficacy of the belinostat/doxorubicin combination treatment. The secondary objectives were to examine the time to response, duration of response, and survival following treatment with belinostat/doxorubicin, disease control rate (complete response (CR), partial response (PR), and stable disease (SD)), and pharmacokinetics (PK).

## 2. Materials and Methods

This study was an open-label multicentre, dose escalation phase I/II study (ClinicalTrials.gov: NCT00878800). It was conducted at 3 sites (Aarhus University Hospital, Department of Oncology, Denmark; Royal Marsden Hospital, NHS Trust, Sutton, Surrey, UK; and Herlev Hospital, Department of Oncology, Denmark). The trial was conducted in accordance with the Declaration of Helsinki and ICH-GCP. It was approved by local regulatory authorities and by independent ethics committees, EudraCT number 2006-004345-42. All participating patients provided written informed consent before any study procedures were performed.

### 2.1. Eligibility Criteria

Eligibility criteria were as follows: patients 18 years or older and giving informed consent; performance status (ECOG) ≤2; life expectancy of at least 3 months; acceptable liver, renal, coagulation, and bone marrow function; serum potassium within normal range; and acceptable coagulation status. Eligibility criteria for dose escalation phase I part: patients with histological or cytological confirmed solid tumours (including sarcomas), with no known curative therapy. Eligibility criteria for MTD expansion phase II part: patients with an established diagnosis of STS in need of first-line chemotherapy and with measurable disease.

### 2.2. Main Exclusion Criteria

 Main exclusion criteria were investigational treatment within 4 weeks prior to study treatment; prior anticancer therapy within the last 3 weeks; coexisting significant medical condition or active infection; concurrent second malignancy; history of hypersensitivity to doxorubicin; pregnancy. Main exclusion criteria for dose escalation phase I part: more than two prior doses of anthracycline or more than three prior lines of chemotherapy given for metastatic disease. Main exclusion criteria for MTD expansion phase II part: prior chemotherapy and LVEF below normal range (45% by MUGA).

### 2.3. Study Design

Belinostat was administered as a 30-minute intravenous infusion every 24 hours on days 1–5 and on day 5 in combination with doxorubicin, in a 3-week schedule, and doxorubicin was given after belinostat. The dose escalation schedule was as follows: belinostat 600 mg/m^2^ and 50 mg/m^2^ doxorubicin (cohort 1), 600 mg/m^2^ and 75 mg/m^2^ doxorubicin (cohort 2), 800 mg/m^2^ and 75 mg/m^2^ doxorubicin (cohort 3), and 1000 mg/m^2^ and 75 mg/m^2^ doxorubicin (cohort 4). See Table 1 for the dose escalation schedule.

Completion of 6 cycles of belinostat/doxorubicin was considered the standard trial duration for each patient. Patients who achieved clinical benefit from the treatment (objective response or stable disease) could continue belinostat treatment beyond cycle 6. If the maximum cumulative dose of doxorubicin 450 mg/m^2^ was reached, the treatment would continue with belinostat as monotherapy. In the phase I part, at least 3 patients were treated at each dose level. If a DLT was found in one of 3 patients, the cohort was expanded to a total of 6 patients. When the MTD was established, a total of 20 patients with STS were to be enrolled in the phase II part of the study at the MTD dose level to examine efficacy and safety in this specific patient population. The phase II part of the trial would be stopped if no more than 2 responses were seen among the 20 patients within their first 2 treatment cycles.

### 2.4. Safety and Tolerability

Patients had medical history, concomitant medication, physical exam, performance status, vital signs, electrocardiogram (ECG), haematology and chemistry profiling, urine analysis, chest X-ray, and tumour evaluation, investigated at baseline. MUGA scan and troponin levels were performed at baseline, at day 1 of every 2nd cycle, and at the end of treatment. Safety assessments including blood test and clinical assessments were performed on days 1, 2, 3, 4, 5, 8, and 15 during cycles 1 and 2 and weekly from cycle 3 onwards and every second week for a minimum of 4 weeks after the end of treatment.

In cycles 1 and 2, ECG was performed before treatment and within 1 h after treatment with each administration instance of belinostat and doxorubicin. In cycles 3–6 one ECG was performed before treatment and within 1 h after treatment with the belinostat administration.

Adverse events were graded using the NCI common terminology criteria for adverse events (NCI-CTCAE) version 3.0. A DLT was defined as the presence of one of the following toxicities during the first cycle of treatment: absolute neutrophil count <0.5 *∗* 10^9^/L lasting for ≥7 days; absolute neutrophil count <0.5 *∗* 10^9^/L with fever >39°C; platelet count <25 *∗* 10^9^/L; and any other drug-related grade 3 or 4 nonhaematological toxicity (with the exception of alopecia, brief nausea and vomiting, diarrhoea, rash, arthralgia, and myalgia). If, despite optimal treatment, grade 3 nausea and/or vomiting persisted or if grade 4 diarrhoea in spite of standard therapeutic measures persisted or there was inability to tolerate a full 5-day dosing cycle due to toxicity or any drug-related adverse events resulting in more than a 14-day treatment delay, these were included in the DLT definition.

### 2.5. Pharmacokinetic Analyses

The following PK parameters were determined: elimination half-life (*t*_1/2_), maximum concentration (*C*_max_), time to maximum concentration (*t*_max_), area under the curve (AUC_0−*t*_last__ and AUC_*∞*_), elimination rate constant (*λz*), and volume of distribution (*V*_c_ and *V*_ss_). Blood sampling for belinostat alone was performed on cycle 1 day 4 (before treatment and 0 min, 5 min, 15 min, 30 min, 1 h, 2 h, 2 h 15 min, 2 h 30 min, 3 h, 4 h, 6 h, 8 h, and 24 h after treatment). Blood sampling for belinostat/doxorubicin was performed on cycle 1 day 5 (before treatment and 0 min, 5 min, 15 min, 30 min, 1 h, 2 h, 2 h 15 min, 2 h 30 min, 3 h, 4 h, 6 h, 8 h, and 24 h after treatment).

### 2.6. Tumour Response

Patients were evaluated clinically after each treatment cycle and radiologically at baseline and after every two cycles with computed tomography (CT) until PD. A confirmatory scan was obtained 4 weeks following initial documentation of an objective response (CR and PR). Response and progression were evaluated according to the Response Evaluation Criteria in Solid Tumours (RECIST, version 1.0) [12]. Relevant tumour markers such as CA125, CEA, and PSA were used to assess the effect of belinostat/doxorubicin on the various tumour types.

### 2.7. Statistics

Descriptive statistics (incidence and confidence intervals) were used to summarize the number of patients exhibiting objective response (OR) or stable disease (SD). Data are expressed as a proportion + 95% confidence interval.

## 3. Results

A total of 25 patients were enrolled and treated in the dose escalation phase I part of the study and a total of 16 patients were enrolled and treated in the MTD expansion phase II part. Four patients with STS in cohort 4 were treated at the MTD dose level and therefore these patients were included in the MTD phase II part. See Table 2 for demographics.

The patients received a median of 4 cycles (range 1–19) of belinostat and 4 cycles (range 1–9) of doxorubicin and 6 patients continued with single-agent belinostat.

### 3.1. Dose Limiting Toxicities and Maximum Tolerated Dose

In the dose escalation phase I part, one event of CTC grade 3 rash/hand and foot syndrome, in cohort 3, day 22, was considered as a DLT; the patient had progressive disease and went off the study after day 2 of cycle 2.

The MTD was established at the highest tested dose level: belinostat 1000 mg/m^2^/d and doxorubicin 75 mg/m^2^ (cohort 4).

### 3.2. Safety, Tolerability, and Dose Reduction

A total of 1059 treatment emergent adverse events were recorded in the study; 63% of these adverse events (AE) were assessed as related to study drugs. The most frequent AEs across all dosing groups were fatigue (95%), nausea (76%), alopecia (63%), vomiting (59%), constipation (54%), neutropenia (54%), dyspnoea (54%), decreased appetite (54%), headache (44%), and injection site reaction (39%). See Table 3 for related CTC grade 3 and 4 adverse events.

The most frequent AEs occurring in the MTD expansion phase II part were fatigue (94%), nausea (88%), and alopecia (81%).

Three AEs in 2 patients led to study discontinuation: febrile neutropenia (grade 4) and fatigue (grade 3) both recorded in 1 patient and an episode of neutropenic sepsis (grade 4) recorded in another patient. Both patients were treated at the 800 mg belinostat and 75 mg doxorubicin dosing level.

A total of 34 SAEs in 23 patients were recorded. One patient died of disease progression, unrelated to study drug. Six SAEs in 6 patients were CTC grade 4, 18 SAEs in 14 patients were CTC grade 3, and 9 SAEs in 8 patients were CTC grade 2. The 6 grade 4 SAEs were all considered treatment related and were febrile neutropenia (3 pts (1 patient cohort 2 day 17, 1 patient cohort 3 day 15, and 1 patient MTD cohort day 37)), neutropenia (2 patients, both patients MTD cohort day 15 and day 148), and neutropenic sepsis (1 patient cohort 3 day 15).

Of the 20 patients with sarcomas treated at the MTD dose, 12 patients (60%) experienced CTC grade 4 neutropenia. A total of 11 patients (55%) required a dose reduction of both belinostat and doxorubicin and 2 of doxorubicin only. Two patients discontinued treatment after completing cycle 1 due to progressive disease.

### 3.3. Pharmacokinetics

Following a 30-minute IV infusion of belinostat at 600, 800, and 1000 mg/m^2^ on day 4 and coadministration with 50 or 75 mg/m^2^ doxorubicin on day 5, plasma concentrations of belinostat declined in an apparent multiphasic manner, with a rapid distribution/elimination phase followed by a longer terminal elimination phase. The geometric mean apparent elimination half-life (*t*_1/2_) appeared dose independent ranging from approximately 1.9 to 4.8 hours with belinostat plasma concentrations generally quantifiable up to 8–24 hours after the start of the infusion. See Table 4 for pharmacokinetics.

Regarding the in-between-patient variability (16.2 to 43.2% for AUC_0−*t*_last__ and 13.6 to 69.2% for *C*_max_) belinostat appeared to exhibit dose-proportional pharmacokinetics across the dose range studied. In addition, there was no evidence that coadministration of doxorubicin had any effect on belinostat exposure.

The metabolite doxorubicinol was steadily formed from parent drug with median *t*_max_ ranging from 2.0 to 4.9 hours after start of doxorubicin infusion compared to median *t*_max_ of 0.24 to 0.5 hours for doxorubicin. The exposure of doxorubicin and its metabolite doxorubicinol showed dose-proportional increase between the 50 and 75 mg/m^2^ doxorubicin dose levels when coadministered with 600, 800, and 1000 mg/m^2^ belinostat on day 5. The geometric mean *t*_1/2_ of doxorubicin was similar for all doses ranging from 7.6 to 10.8 hours.

Dose normalised AUC_0−*t*_last__ and *C*_max_ appeared relatively consistent across treatment groups on both days 4 and 5 indicating that doxorubicin exhibited linear pharmacokinetics results between 50 and 75 mg/m^2^ doses; coadministration of belinostat had no effect on doxorubicin exposure.

### 3.4. Tumour Response and Survival Analysis

See Figures 2, 3, and 4.

Dose escalation phase I part (*n* = 25): two patients (one with the diagnosis of squamous cell carcinoma of the cervix (belinostat: 600 mg/m^2^/doxorubicin: 75 mg/m^2^) and one retroperitoneal angiosarcoma (belinostat: 1000 mg/m^2^/doxorubicin: 75 mg/m^2^)) had PR as objective response, corresponding to a response rate of 8% (95% CI, 1–26%); time to progression for these patients was 9.6 and 7.9 months, respectively. Median duration of response was 3.9 months. SD was observed in 16 (64%) patients; 7 (28%) had PD. Median time to progression was 3.7 months (95% CI, 3.0–5.6 months). 18 patients had disease control corresponding to a disease control rate of 72% (95% CI, 51–88%).

MTD expansion phase II part (*n* = 16): two patients out of 16 had an objective response (pleomorphic sarcoma (CR) and leiomyosarcoma (PR)), leading to a response rate of 13% (95% CI, 2–38%), and one of these responses occurred after cycle 2. Median duration of response was 7.9 months. SD was observed in 9 (56%) patients and 5 (31%) had PD. Median time to progression was 6.0 months (95% CI, 1.6–9.7 months). Eleven patients had disease control, corresponding to a disease control rate of 69% (95% CI, 41–89%).

**Figure 1:**
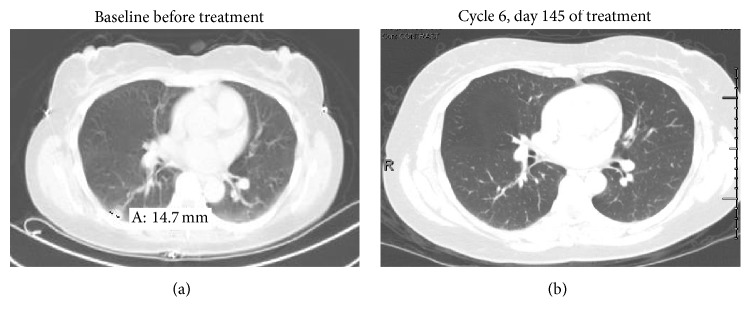
Imaging of patient with complete remission.

**Figure 2:**
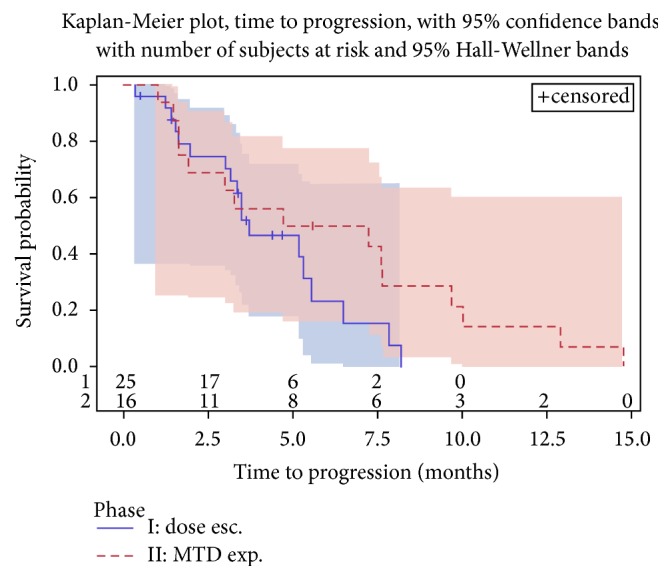
Time to progression for phase I and phase II.

**Figure 3:**
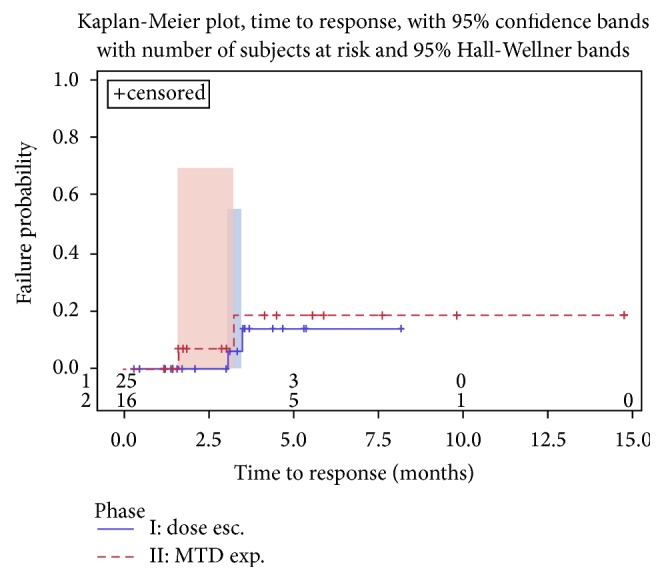
Time to response for phase I and phase II.

**Figure 4:**
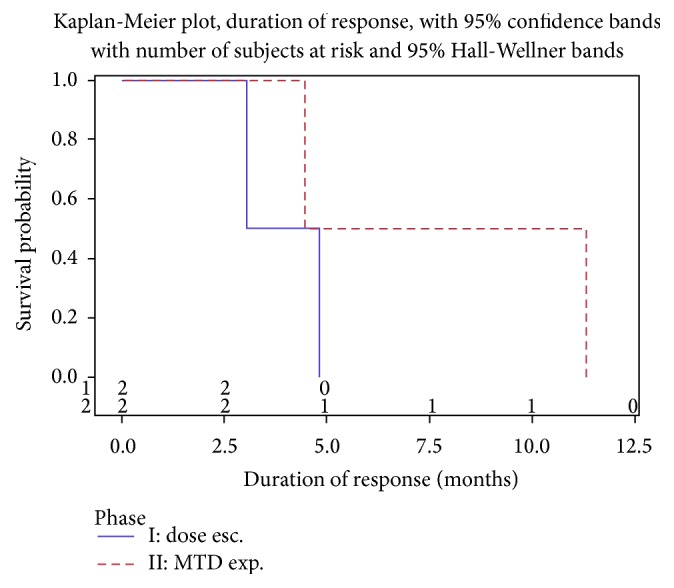
Duration of response for phase I and phase II.

In the MTD expansion phase II part the patient who experienced a complete response was a female, age 38, with a diagnosis of leiomyosarcoma. She experienced a partial response after 49 days and a complete response after 145 days and had progressive disease after 19 treatment cycles, on day 345. Serial imaging for this patient is displayed in Figure 1.

The trial stopping rule defined that if no more than 2 responses were seen among the first 20 patients within the first 2 treatment cycles, the study would be stopped. With only one patient experiencing an early response, the study was stopped for accrual based on data from 4 sarcoma patients in the dose escalation phase and 16 patients at the MTD level.

### 3.5. Reasons for Discontinuation

PD was the most frequent reason for discontinuation (73%), 4 patients withdrew consent, 3 patients discontinued due to AEs, 3 patients discontinued for other reasons, and 1 patient died due to PD which was evaluated as being unrelated to treatment by the investigator.

## 4. Discussion

HDAC inhibitors are a new class of anticancer agents and are being evaluated for the treatment of a variety of malignancies. Soft tissue sarcoma is a heterogeneous group of tumours and patients with metastatic disease have a poor outcome. Therefore, there is an urgent medical need to develop new treatment options for this particular group of patients.

As previously mentioned preclinical data have shown a strong synergistic effect of combining belinostat and doxorubicin in the sarcoma cell lines SaOS2 and U2OS and clinical data from a phase I trial using belinostat as monotherapy showed prolonged SD in two pts with STS for 7 and 14 months, respectively [10]. In contrast other studies have shown that there is little benefit in using belinostat as monotherapy and that it should preferably be used in combination with other agents [13–15]. The recognized ability of HDAC inhibitors to affect the expression of genes involved in DNA-damage is thought to be the cause of the synergy with cytotoxic agents that specifically determine DNA-damage, such as doxorubicin, a topoisomerase II inhibitor [16, 17]. Currently doxorubicin is the most effective drug in the treatment of metastatic STS. Therefore, these data indicated a strong rationale for conducting a study combining belinostat and doxorubicin.

Our study demonstrated that belinostat 1000 mg/m^2^/d in combination with doxorubicin 75 mg/m^2^ can be safely administered to patients with advanced solid tumours. The combination was generally well tolerated with the most common AEs being fatigue, nausea, alopecia, vomiting, constipation, and neutropenia; however neutropenia (all grades) was observed in 54% of the patients across the different dosing levels and 55% of the patients in the MTD expansion phase II part experienced a dose reduction due to adverse events.

Two patients experienced a partial response in the dose escalation phase I part, whereas 2 patients in the MTD expansion phase II part also experienced responses, one having an impressive complete remission and one having partial response. The activity of the combination of belinostat and doxorubicin in soft tissue sarcoma in terms of response rate was 13%, which is in line with already published data on single-agent doxorubicin, where a response rate of 14% is reported [6]; however a significant number of patients (55%) treated at the MTD dose level experienced stable disease, of whom 7 patients were treated for 6 or more cycles.

There is no standard treatment for advanced chondrosarcomas. Based on the preclinical study with activity of belinostat + doxorubicin in bone derived sarcomas combined with a positive observation of stable disease for 4 courses in one patient with chondroblastic chondrosarcoma in the dose escalation phase I part, we accepted the 3 patients with chondrosarcomas for the phase II trial. The patients were therefore included in the analysis. We did not observe any objective response in these 3 patients; one patient had progression after 2 courses, one patient had stable disease and progression after 4 courses, and the last patient had stable disease for 8 courses and then progression. Therefore this subgroup of patients did not influence the overall moderate response rate much.

Certain aspects in the setup of this study and in study patient characteristics warrant further mentioning as potential reasons for influencing the moderate response rate: the use of G-CSFs was not permitted in the study where neutropenia (all grades) was as mentioned above observed in 54% of all patients. Eleven patients (55%) from the MTD expansion phase 2 part had a dose reduction in both belinostat and doxorubicin due to adverse events. The dose reduction in 55% of the patients could be a potential cause for the moderate response. Neutropenia (all grades) has been reported in 37% with single-agent doxorubicin which is less than in our study; in that trial G-CSF was also allowed and could influence the incidence of neutropenia [6].

Three of the 4 responders were diagnosed with STS and all of these were treated at the highest dose level (belinostat: 1000 mg/m^2^/doxorubicin: 75 mg/m^2^). If calculating the response rate in the group of patients with STS that received the highest dose level, the response rate is 15% and if excluding the chondrosarcomas, the response rate is 18%.

The study was as previously mentioned designed with a stopping rule based on response rate. Recent soft tissue sarcoma studies have used stopping rules based on progression-free rate, acknowledging the benefit of disease stabilization in this disease [1, 18, 19] and SD was observed in 11 (55%) with STS treated at the MTD dose level.

## 5. Conclusions

Belinostat is approved in the USA for the treatment of relapsed/refractory peripheral T-cell lymphoma (PTCL) and although one must conclude that there was no clear evidence of synergy between belinostat and doxorubicin in terms of objective tumour shrinkage in soft tissue sarcomas, median time to progression in this study was 6 months, which is superior to some reports of first-line single-agent doxorubicin in soft tissue sarcoma [20, 21].

## Figures and Tables

**Table 1:** Dose escalation scheme for the combination treatment with belinostat and doxorubicin.

Phase	Purpose	Eligibility	Cohort	Bel (mg/m^2^)	Dox (mg/m^2^)	*N*
I	Dose escalation	pts with STS and other solid tumours	1	600	50	3
			2	600	75	7
			3	800	75	9
			4	1000	75	6

II	MTD expansion	pts with STS only	Expansion 1	MTD	MTD	20 (16 plus 4 from cohort 4)
			Expansion 2	MTD	MTD	0

STS: soft tissue sarcoma.

MTD: maximum tolerated dose.

**Table 2:** Demographics.

Characteristics	Patients	
Total number of patients	41	

Age (years)		
Mean	57.1	
Range	(30–71)	

Gender		
Female	15 (37%)	
Male	26 (63%)	

ECOG performance status	*N*: 41	

0	23	
1	14	
2	4	

Tumour type	DE (*N*: 25)	MTD (*N*: 16)

*Solid tumours, total*	15	0
Colorectal adenocarcinoma	1	0
Non-small cell lung cancer	1	0
Renal pelvis carcinosarcoma	1	0
Pancreas adenocarcinoma	1	0
Cholangiocarcinoma	1	0
Cervix cell carcinoma	1	0
Stromal tumour of unknown	1	0
Anal planocellular carcinoma	1	0
Ocular malignant melanoma	3	0
Bladder carcinoma	1	0
Renal carcinoma	1	0
Squamous cell carcinoma	1	0
Thyroid carcinoma	1	0

*Sarcomas, total*	10	16
Liposarcoma	2	3
Leiomyosarcoma	2	5
Chondrosarcoma	1	3
Angiosarcoma	1	1
Myxofibrosarcoma	1	1
Undif pleomorphic sarcoma	1	1
Rhabdomyosarcoma	1	0
Fibrosarcoma	1	0
Myogenic sarcoma	0	1
Synovial sarcoma	0	1

DE: dose escalation phase I study.

MTD: maximum tolerated dose phase II study.

**Table 3:** Grade 3 and 4 treatment-related adverse events occurring in both the DE and MTD part of the study.

Adverse event, *N* (%)	Cohort 1 (*N*: 3) Belinostat 600 (mg/m^2^) Doxorubicin 50 (mg/m^2^)	Cohort 2 (*N*: 7) Belinostat 600 (mg/m^2^) Doxorubicin 75 (mg/m^2^)	Cohort 3 (*N*: 9) Belinostat 800 (mg/m^2^) Doxorubicin 75 (mg/m^2^)	Cohort 4 (*N*: 6) Belinostat 1000 (mg/m^2^) Doxorubicin 75 (mg/m^2^)	MTD (*N*: 16) Belinostat 1000 (mg/m^2^) Doxorubicin 75 (mg/m^2^)
*Fatigue*					
Grade 3	0 (0%)	0 (0%)	2 (22%)	0 (0%)	3 (19%)
Grade 4	0 (0%)	0 (0%)	1 (11%)	0 (0%)	0 (0%)

*Neutropenia*					
Grade 3	0 (0%)	3 (43%)	2 (22%)	1 (17%)	4 (25%)
Grade 4	0 (0%)	3 (43%)	0 (0%)	1 (17%)	5 (31%)

*Anaemia*					
Grade 3	0 (0%)	0 (0%)	0 (0%)	0 (0%)	2 (13%)
Grade 4	0 (0%)	0 (0%)	0 (0%)	0 (0%)	0 (0%)

*Febrile neutropenia*					
Grade 3	0 (0%)	1 (14%)	0 (0%)	0 (0%)	2 (13%)
Grade 4	0 (0%)	1 (14%)	1 (11%)	0 (0%)	1 (6%)

*Mucosal inflam*					
Grade 3	0 (0%)	0 (0%)	0 (0%)	0 (0%)	1 (6%)
Grade 4	0 (0%)	0 (0%)	0 (0%)	0 (0%)	0 (0%)

*Abdominal abscess*					
Grade 3	0 (0%)	1 (14%)	0 (0%)	0 (0%)	0 (0%)
Grade 4	0 (0%)	0 (0%)	0 (0%)	0 (0%)	0 (0%)

*Bacteraemia*					
Grade 3	0 (0%)	1 (14%)	0 (0%)	0 (0%)	0 (0%)
Grade 4	0 (0%)	0 (0%)	0 (0%)	0 (0%)	0 (0%)

*Prolonged QT*					
Grade 3	0 (0%)	1 (14%)	0 (0%)	0 (0%)	0 (0%)
Grade 4	0 (0%)	0 (0%)	0 (0%)	0 (0%)	0 (0%)

*Neutrophil count*					
Grade 3	0 (0%)	1 (14%)	0 (0%)	3 (50%)	0 (0%)
Grade 4	0 (0%)	0 (0%)	0 (0%)	0 (0%)	0 (0%)

*Decreased appetite*					
Grade 3	0 (0%)	0 (0%)	0 (0%)	0 (0%)	1 (6%)
Grade 4	0 (0%)	0 (0%)	0 (0%)	0 (0%)	0 (0%)

*Rash*					
Grade 3	0 (0%)	0 (0%)	1 (11%)	0 (0%)	0 (0%)
Grade 4	0 (0%)	0 (0%)	0 (0%)	0 (0%)	0 (0%)

*Neutropenic sepsis*					
Grade 3	0 (0%)	0 (0%)	0 (0%)	0 (0%)	0 (0%)
Grade 4	0 (0%)	0 (0%)	1 (11%)	0 (0%)	0 (0%)

*Hypersensitivity*					
Grade 3	0 (0%)	0 (0%)	1 (11%)	0 (0%)	0 (0%)
Grade 4	0 (0%)	0 (0%)	0 (0%)	0 (0%)	0 (0%)

*Headache*					
Grade 3	0 (0%)	0 (0%)	1 (11%)	0 (0%)	0 (0%)
Grade 4	0 (0%)	0 (0%)	0 (0%)	0 (0%)	0 (0%)

*Leukopenia*					
Grade 3	0 (0%)	0 (0%)	0 (0%)	1 (17%)	0 (0%)
Grade 4	0 (0%)	0 (0%)	0 (0%)	0 (0%)	0 (0%)

*Pneumonia*					
Grade 3	0 (0%)	0 (0%)	0 (0%)	1 (17%)	0 (0%)
Grade 4	0 (0%)	0 (0%)	0 (0%)	0 (0%)	0 (0%)

*Wound infection*					
Grade 3	0 (0%)	0 (0%)	0 (0%)	1 (17%)	0 (0%)
Grade 4	0 (0%)	0 (0%)	0 (0%)	0 (0%)	0 (0%)

*Neutrophil count decreased*					
Grade 3	0 (0%)	0 (0%)	0 (0%)	0 (0%)	0 (0%)
Grade 4	0 (0%)	0 (0%)	0 (0%)	1 (17%)	3 (19%)

*Platelet count decreased*					
Grade 3	0 (0%)	0 (0%)	0 (0%)	0 (0%)	0 (0%)
Grade 4	0 (0%)	0 (0%)	0 (0%)	1 (17%)	0 (0%)

*Blood sodium decreased*					
Grade 3	0 (0%)	0 (0%)	0 (0%)	0 (0%)	0 (0%)
Grade 4	0 (0%)	0 (0%)	0 (0%)	0 (0%)	1 (6%)

*Diarrhoea*					
Grade 3	0 (0%)	1 (14%)	0 (0%)	0 (0%)	0 (0%)
Grade 4	0 (0%)	0 (0%)	0 (0%)	0 (0%)	0 (0%)

DE: dose escalation phase I study.

MTD: maximum tolerated dose phase II study.

**Table 4:** Pharmacokinetic parameters of belinostat (Bel) following administration of Bel alone (cycle 1 day 4) or in combination with doxorubicin (cycle 1 day 5).

Occasion	600 mg/m^2^ Bel + 50 mg/m^2^ doxorubicin		600 mg/m^2^ Bel + 75 mg/m^2^ doxorubicin		800 mg/m^2^ Bel + 75 mg/m^2^ doxorubicin		1000 mg/m^2^ Bel + 75 mg/m^2^ doxorubicin	
	Day 4	Day 5	Day 4	Day 5	Day 4	Day 5	Day 4	Day 5
	(*N* = 3)	(*N* = 3)	(*N* = 7)	(*N* = 6)	(*N* = 8)	(*N* = 6)	(*N* = 21)	(*N* = 19)
AUC_0–*t*_last__ (ng·h/mL)	10900 (16.2)	10400 (22.3)	9670 (40.0)	10400 (24.7)	12200 (28.5)	14600 (26.0)	23100 (35.4)	22100 (43.2)
*C*_max_ (ng/mL)	21100 (13.6)	18300 (36.2)	16800 (69.2)	17400 (45.1)	21400 (44.9)	26400 (30.0)	41100 (35.0)	37000 (59.1)
*t*_max_^a^ (h)	0.533 (0.500–0.550)	0.567 (0.550–0.583)	0.567 (0.500–0.783)	0.517 (0.467–0.667)	0.533 (0.500–0.583)	0.533 (0.483–0.583)	0.617 (0.500–0.750)	0.633 (0.517–1.60)
*t*_last_^a^ (h)	24.5 (22.4–24.6)	17.0 (17.0–20.1)	24.0 (8.55–24.5)	24.5 (16.2–24.7)	23.7 (8.47–24.6)	15.1 (8.50–24.6)	8.65 (1.05–24.0)	8.72 (8.50–24.7)
AUC_0–*t*_last__ (norm)	18.2 (16.2)	17.3 (22.3)	16.1 (40.0)	17.3 (24.7)	15.3 (28.5)	18.2 (26.0)	23.1 (35.4)	22.1 (43.2)
*C*_max_ (norm)	35.2 (13.6)	30.5 (36.2)	28.0 (69.2)	28.9 (45.1)	26.8 (44.9)	32.9 (30.0)	41.1 (35.0)	37.0 (59.1)
*t*_1/2_ (h)	NC (NC)	2.53^e^ (NC)	3.77^b^ (136.4)	4.83^b^ (45.7)	1.91^c^ (129.8)	2.14^c^ (114.9)	1.48^d^ (71.3)	2.13 (119.4)
CL (L/h)	NC (NC)	106^e^ (NC)	129^b^ (61.8)	111^b^ (12.1)	127^c^ (40.5)	91.0^c^ (18.1)	81.6^d^ (39.9)	85.2 (41.0)
*V*_ss_ (L)	NC (NC)	72.8^e^ (NC)	149^b^ (365.2)	90.8^b^ (7.1)	107^c^ (195.5)	58.3^c^ (29.5)	51.0^d^ (45.1)	57.6 (61.8)

*N*: number of patients studied.

^a^Median (min–max).

(norm): normalised for dose.

NC: not calculable.

^b^ *N*: 3.

^c^ *N*: 4.

^d^ *N*: 18.

^e^ *N*: 1; individual parameter presented.
